# WAUC: A Multi-Modal Database for Mental Workload Assessment Under Physical Activity

**DOI:** 10.3389/fnins.2020.549524

**Published:** 2020-12-01

**Authors:** Isabela Albuquerque, Abhishek Tiwari, Mark Parent, Raymundo Cassani, Jean-François Gagnon, Daniel Lafond, Sébastien Tremblay, Tiago H. Falk

**Affiliations:** ^1^Institut National de la Recherche Scientifique - Énergie, Matériaux et Télécommunications, Université du Québec, Montréal, QC, Canada; ^2^Thales Digital Solutions Inc., Québec City, QC, Canada; ^3^École de Psychologie, Université Laval, Québec City, QC, Canada; ^4^PERFORM Centre, Concordia University, Montréal, QC, Canada

**Keywords:** mental workload, operator functional state, workload assessment, wearable sensors, multi-modal database, ambulant subjects

## Abstract

Assessment of mental workload is crucial for applications that require sustained attention and where conditions such as mental fatigue and drowsiness must be avoided. Previous work that attempted to devise objective methods to model mental workload were mainly based on neurological or physiological data collected when the participants performed tasks that did not involve physical activity. While such models may be useful for scenarios that involve static operators, they may not apply in real-world situations where operators are performing tasks under varying levels of physical activity, such as those faced by first responders, firefighters, and police officers. Here, we describe WAUC, a multimodal database of mental Workload Assessment Under physical aCtivity. The study involved 48 participants who performed the NASA Revised Multi-Attribute Task Battery II under three different activity level conditions. Physical activity was manipulated by changing the speed of a stationary bike or a treadmill. During data collection, six neural and physiological modalities were recorded, namely: electroencephalography, electrocardiography, breathing rate, skin temperature, galvanic skin response, and blood volume pulse, in addition to 3-axis accelerometry. Moreover, participants were asked to answer the NASA Task Load Index questionnaire after each experimental section, as well as rate their physical fatigue level on the Borg fatigue scale. In order to bring our experimental setup closer to real-world situations, all signals were monitored using wearable, off-the-shelf devices. In this paper, we describe the adopted experimental protocol, as well as validate the subjective, neural, and physiological data collected. The WAUC database, including the raw data and features, subjective ratings, and scripts to reproduce the experiments reported herein will be made available at: http://musaelab.ca/resources/.

## 1. Introduction

The ability of humans to perform activities in an effective and sustainable way is crucial in situations where tasks are not fully automatic. In many scenarios, human performance might be safety-critical for human lives, such as in the case of tasks performed by aircraft pilots, firefighters, and first responders. In these cases, monitoring and quantifying the current capability of a subject to correctly perform a task may be critical to prevent accidents and, consequently, save lives. In this context, the Operator Functional State (OFS) (Hockey, 2003a) research framework can be used to breakdown the relationship between human performance and the level of difficulty of the respective task (Ting et al., 2009). According to Hockey (2003b), OFS can be defined as “the variable capacity of the operator for effective task performance in response to task and environmental demands, and under the constraints imposed by cognitive and physiological processes that control and energize behavior.” The operator functional state can be thus seen as the resulting interaction between a subject and a task, given specific environmental (e.g., noise, movement, and temperature) and psychophysiological (e.g., sleep loss, illness, fatigue, and anxiety) conditions. While the interplay between human, task, and environment influences task performance, as a compensatory mechanism attempts to sustain task performance levels, this change of resource allocation might increase psychophysiological activation (Ting et al., 2009). The capability of reliably monitoring OFS is key to constraining work shifts and adapting task demand levels, thus ensuring that operators are safely and optimally performing the designated tasks (Wilson and Russell, 2003a).

OFS is also defined as the processes that mediate task performance under stress and high workload (Hockey, 2003a). In this work, we are interested in the impact of mental workload (MW) on the operator functional state. Across several definitions in the literature, MW can be summarized as a construct that encompasses one's capability of performing a task along with the mental strain required for performing it under specific environmental conditions (Cain, 2007). The interest on studying this specific aspect that influences OFS stems from the importance of maintaining its balance during task execution. In case the operator needs to employ high levels of mental resources in order to achieve a required task performance for a long time, this might increase fatigue levels to such a point that the operator is no longer able to successfully perform the task. On the other hand, if the task is not demanding enough, it can lead to boredom and lack of engagement, which could also affect the operator's performance (Wilson and Russell, 2003a; Jasper et al., 2016). However, devising an objective strategy to assess MW is still an open challenge. One of the main reasons is its subjectivity, as different factors such as previous experience and temporal pressure might affect how each subject perceives the level of difficulty when performing a task (Charles and Nixon, 2019).

Mental workload can be assessed via subjective ratings, task performance outcomes, and psychophysiological measures. Each method considers different inputs and presents different time resolutions. Among those, strategies based on monitoring psychophysiological signals collected with wearable devices present the best temporal resolution, as they may capture OFS changes even before they are reflected in task performance (Ting et al., 2009). In the literature, clinical-grade devices are frequently employed to monitor psychophysiological responses (Yin and Zhang, 2017; Hefron et al., 2018). However, these devices usually require a long time to be setup, are not comfortable to be worn for extended periods, and might not allow the monitored subject to walk freely to perform their tasks. Thus, when considering real-world scenarios, where it is not possible to use clinical-grade devices to collect the required data, the use of wearable technology becomes key to enable MW monitoring. A further barrier to the wide deployment of MW models in real-world scenarios lies in the mismatch between training and testing conditions, as the former have typically relied on static subjects (usually sitting on a chair) performing tasks that do not demand intensive body movement. Representative examples include tasks such as the N-back (Milner, 1998) and the Cabin Air Management System (Sauer et al., 2000). As such, current models do not explicitly take into account factors such as movement artifacts and the interplay between physical activity and MW, and thus it is not possible to directly apply them in situations that involve ambulant subjects.

In order to decrease the gap between current research on MW assessment based on psychophysiological signals and real-world applications, here we describe a dataset collected using consumer-grade wearable devices in conditions that combine manipulation of MW levels with different levels of physical strain. The study involved 48 subjects and six neural and physiological modalities were acquired (electroencephalography, electrocardiography, breathing rate, skin temperature, galvanic skin response, and blood volume pulse), in addition to 3-axis accelerometry. Moreover, after each experimental session, subjective ratings of MW using the NASA Task Load Index questionnaire (Hart and Staveland, 1988) and physical fatigue using the Borg Scale (Borg, 1982) were collected.

We focus on providing resources for allowing the development of different strategies for assessing MW. By monitoring psychophysiological responses to tasks that gauge distinct levels of MW, it is possible to employ the collected signals to compute features that act as a proxy to quantify how much the OFS was affected by the respective change in the task. More specifically, we developed an experimental protocol using the Multi-Attribute Task Battery II (MATB-II) (Santiago-Espada et al., 2011) in which participants performed a cognitive task under two levels of MW (low, high) and under three levels of physical activity (no, medium, high) by either walking/running on a treadmill or riding a stationary bike. Recent works (e.g., Wilson and Russell, 2003b; Cassenti et al., 2010) have shown that the MATB-II better elicits MW than tasks typically reported in the literature, such as the N-back task (Milner, 1998), mental rotation (Johnson, 1990), and visual search (Shepard and Metzler, 1971). This experimental design allows investigating questions that remain elusive in the MW assessment literature, such as the interplay between different modalities, and the impact of increased physical activity and movement on MW correlates in terms of added artifacts, as well as what additional mental resources are drawn by the physical activity.

In the following, we summarize the main contributions of the WAUC dataset:

- Experimental setting more closely resembling real-world applications where mental and physical workload are simultaneously considered.
- Large number of participants (48) in comparison to similar studies.
- Two different physical activity modulators tested, namely, stationary bike and treadmill.
- Multiple signals modalities are provided, all time-synchronized during the collection process, to allow for multi-modal MW models to be developed.
- Ground-truth values for both mental and physical workload are provided, as well as perceived values measured via subjective ratings.

The remainder of this paper is organized as follows: in section 2, we provide a brief literature review on MW assessment and existing datasets. In section 3, we describe the experimental protocol. In sections 4, 5, respectively, we describe the experiments performed to validate the WAUC dataset and present the results. Conclusions are given in section 6.

## 2. Related Work

In this section, we provide a brief overview of the literature related to the proposed dataset. We describe MW assessment methods based on subjective ratings, as well as methods that utilize neural and physiological data as source of information. Our dataset comprises multiple modalities collected from ambulant subjects, and to the best of our knowledge, no similar experimental setting was previously proposed in the MW assessment literature. Thus, due to lack of closely related work, we decided to highlight in this section previous work that utilized data collected when subjects were performing similar tasks to the ones considered in our experimental protocol. At last, we briefly describe similar datasets that provided multiple neural and physiological modalities but were not proposed with the aim of performing MW assessment.

### 2.1. Subjective Mental Workload Assessment

Given the importance of maintaining balanced levels of MW for successful and safe performance of critical tasks, several works in the literature proposed strategies for assessing this dimension of the OFS. Part of this previous work proposed to tackle the MW assessment problem using subjective measurements collected while the task was being performed. Such methods rely on participants periodically filling in a questionnaire with ratings related to their current OFS. Popular examples are the Subjective Workload Assessment Technique (SWAT) (Reid and Nygren, 1988), the NASA Task Load Index (NASA-TLX) (Hart and Staveland, 1988), and the Modified Cooper–Harper Scale (Wierwille and Casali, 1983). These methods feature a multi-scale grading of multiple MW aspects. One main drawback of using such questionnaires across multiple sessions, however, is that they do not take into account relative changes in the ratings for each time the subject answers the questions. In order to circumvent this issue, Vidullch et al. (1991) proposed the Subjective Workload Dominance (SWORD) technique, which consists of comparing pairs of tasks to build the so-called judgment matrix and then computing a final workload index. Similarly to the SWORD questionnaire, SWAT and NASA-TLX ratings can also be aggregated in order to provide a single workload measure.

Even though the methods based on subjective ratings collected at the same time the task is executed present a low-cost and easy-to-implement alternative to assess MW, this strategy presents critical limitations. As highlighted by Borghini et al. (2014), attending to a secondary rating task might increase the levels of working memory required to perform the main task. Thus, the sole act of filling the questionnaires may be responsible to changes in the reported MW. Moreover, those methods do not allow for continuous MW assessment and have poor temporal resolution. While reducing the intervals at which the operator needs to provide feedback could lead to improved temporal resolution, this may actually increase workload due to the number of interruptions to the task being performed.

### 2.2. Mental Workload Assessment From Neural and Physiological Data

Due to the aforementioned limitations of measuring subjective ratings in real-time during task performance, neural and physiological data collection and analysis have emerged as a promising alternative. Electroencephalography (EEG), for example, has been frequently used to monitor MW mostly due to its high temporal resolution in comparison to other neuroimaging techniques (Teplan et al., 2002). Recent work on EEG-based MW assessment has suggested that using hand-engineered features combined with a classifier to predict MW levels can achieve a satisfactory performance. Zhang et al. (2016) combined EEG spectral features with ensembles of Support Vector Machines to devise subject-specific workload models. Their proposed strategy achieved an average classification performance of 76.7% (5 classes) across seven subjects. The recent literature on EEG-based MW assessment has also been trying to leverage advances in representation learning methods powered by deep neural networks. Almogbel et al. (2018), for example, utilized convolutional neural networks to classify MW states on a task that simulates vehicle driving. Raw EEG was employed and the best model described in the paper obtained 95.3% accuracy on a binary classification task considering the single subject considered in the experimental protocol.

In addition to EEG, physiological responses related to heart rate changes and measured by electrocardiogram (ECG) have also been considered for MW modeling. Heart rate variability (HRV) is frequently employed as a correlate for MW based on cardiac activity. HRV has been shown to successfully capture changes in the sympathetic–parasympathetic balance and to be lowered by an increase in MW levels (Chaumet et al., 2019). In the context of controlling unmanned aerial vehicles, Jasper et al. (2016) verified whether HRV could be used as a predictor of operator MW in this scenario. Each one of the 20 participants simultaneously controlled multiple vehicles while their ECG was monitored. Paired t-tests between HRVs obtained in different parts of the experiment (e.g., planning and executing the task) confirmed the expected effect of lower HRV values as MW increased in terms of required vigilance and situational awareness. In addition to studying the relationship between HRV and MW, Castaldo et al. (2017) also assessed its correlation with performance of repetitive tasks. Their study showed that eight HRV features, such as the mean of RR intervals and approximated entropy, presented a strong correlation with task performance (with *p* > 0.05), which suggests that HRV can also be used as a predictor of how successfully operators will execute a task.

Other studies in the operator functional state monitoring literature have attempted to leverage the complementary between different neural and physiological modalities to achieve improved MW assessment. In their study, Wilson and Russell (2003b) combined EEG, heart rate, eye movement, and respiration rate to model MW elicited using the MATB-II task. Features such as EEG power spectral density and ECG interbeat intervals were used as input to a neural network. The average achieved classification accuracy was 84.3% (high or low MW levels) with a training set, which simultaneously considered data from all subjects. Furthermore, Hogervorst et al. (2014) proposed to use subject-specific models to model MW for the N-back task based on EEG, ECG, skin conductance, respiration, and eye-related measures. Their findings, however, showed that the fusion of different modalities did not improve the performance on MW prediction in comparison to using individual signals.

### 2.3. Physical Activity During EEG Monitoring

The interest on employing EEG-based brain-computer interfaces to real-world applications where ambulant subjects are considered motivated a diverse body of work. Matthews et al. (2008) developed a low-power portable EEG monitoring device capable of long-term signal acquisition. Data were collected while subjects walked on a treadmill at a speed of 2 mph and performed mental tasks such as divide a number by 7 or played a first person video game. A performance of approximately 80% accuracy for binary MW (high or low) assessment was achieved. Snyder et al. (2015) aimed to isolate and investigate the effect of movement artifacts on EEG data. The proposed experimental protocol involved 10 subjects walking on a treadmill at four different speeds. Since the goal was to obtain pure gait-related artifacts, no mental task was performed during the experiment. Their analysis showed that independent component analysis yielded accurate localization for most of the artifacts components. Zink et al. (2016) studied the differences on brain activity due to movement and cognitive effort by proposing an experimental protocol that collected EEG while subjects were cycling on stationary bikes or freely biking. While biking, subjects were asked to perform a three-class oddball auditory task. EEG analysis showed a reduction in the P300 component in cases where subjects were performing physical activity on an unconstrained environment, suggesting that there exists an interplay between increase in cognitive load stemming from freely biking and perceived task difficulty.

### 2.4. Related Datasets

To the best of our knowledge, there is no publicly available multi-modal dataset for MW assessment based on wearables. In contrast, for orthogonal aspects of human cognitive states, such as emotion and affective states, there are a few popular multi-modal datasets with modalities similar to the ones collected here. As examples, we highlight the DEAP (Koelstra et al., 2011) and MAHNOB-HCI (Soleymani et al., 2011) databases. Both analyze human affective states and were recorded while subjects watched videos. In the case of DEAP, music video clips were used. For MAHNOB-HCI, in turn, videos clips were taken from different movies. In both datasets, modalities such as EEG, galvanic skin response (GSR), skin temperature, and breathing rate were made available and time-synchronized. In all cases, subjects were asked to remain still and seated while watching the video clips.

## 3. Methods and Materials

### 3.1. Participants

As the experimental protocol involved sustained physical and mental strain for a considerable period of time, recruited subjects were submitted to a pre-screening process in order to prevent any potential risk during the data collection. Hence, candidates with cardiovascular diseases, neurological disorders, history of feeling dizzy, or fainting were not considered for the experiment. After the screening process, four participants were discarded and 48 were selected. Based on self-identified gender and the assigned physical activity modality (i.e., bike or treadmill) used during the experiment, a total of 22 participants used the treadmill (9 male, 13 female) and 26 performed the experiment using the bike (16 male, 10 female). The average age among the participants was 27.4±6.6 years old. In order to avoid gender bias in our dataset, we intended to have a close number of male and female subjects, however, no candidate was rejected or accepted to participate in our experiment due to gender-related reasons. All participants consented to participating in the study and were remunerated (10 CAD/hour) for the time they spent at the experiment facility. The experimental protocol was approved by the Ethics Review Boards of INRS, Université Laval and the PERFORM Centre (Concordia University), the latter being the location in which data were collected.

Prior to arriving at the experiment facility, participants were advised to wear comfortable sportswear, and to not drink caffeinated beverages for at least 2 h prior to the beginning of the data collection. Before starting the task tutorial, participants were asked to read and sign (in case of agreement) a consent form containing a brief description of the goals of our project and allowing the use and sharing of the collected data for research purposes.

### 3.2. Experimental Protocol

The experimental protocol aimed at simultaneously modulating mental and physical workload. Participants executed mental tasks while performing physical activity. A full factorial (2 MW levels ×3 Physical strain levels) design was employed to capture main effects and interactions. The data collection protocol was preceded by a tutorial to make the participants familiar with the tasks. The tutorial consisted of slides presentation to explain the experimental procedure and the tasks to be executed. Subjects were allowed to take as much time as necessary to go through the tutorial and to ask the experimenters as many questions as needed.

After ensuring the participant understood the tasks to be performed, the next step involved donning the devices. Subjects were first asked to wear a BioHarness 3 chest strap (Zephyr, USA) that integrates the ECG, breathing rate, and acceleration monitoring. Next, an Enobio portable 8-channel wireless EEG headset (Neuroelectrics, Spain) was placed. While electrode connections were checked and calibrated via the device's companion software, a second experimenter placed the E4 wristband (Empatica, USA) responsible to monitor skin temperature, GSR, and blood volume pulse (BVP).

**Figure 1:**
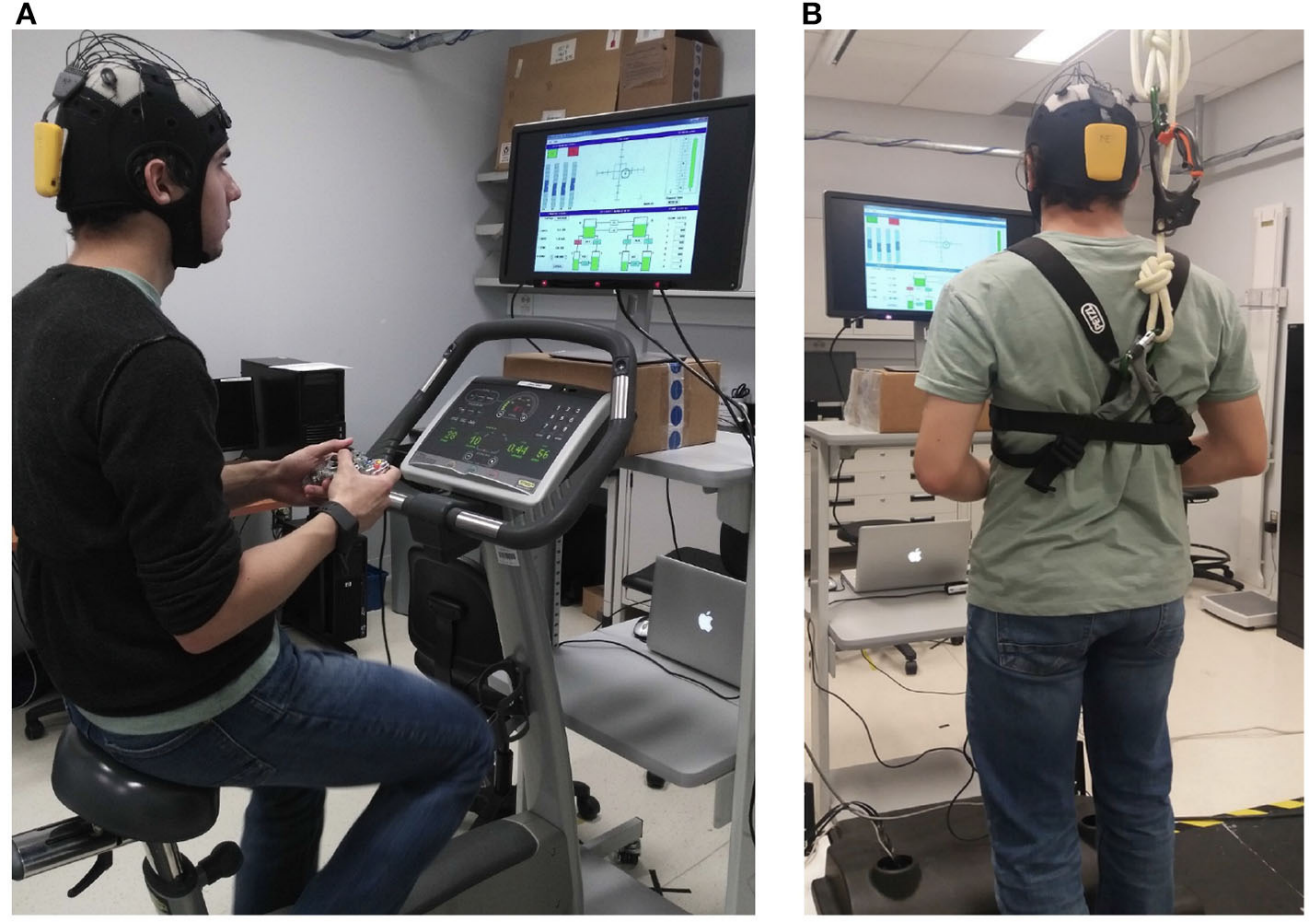
Experimental set-up illustration for **(A)** bike and **(B)** treadmill sessions.

To guarantee participants' safety during the experiment, a safety harness was placed at the participant's chest following the devices placement step mentioned above. This was only the case for the participants assigned to the treadmill task. For those assigned to the stationary bike, they were asked to adjust the seat according to their preference. In all cases, the height of the screen was adjusted lastly according to participants preferences. Figures 1A,B illustrate the experimental layout for the bike and treadmill, respectively, once all devices and safety features are in place. Before starting the data collection, each subject performed a practice session that corresponded to playing MATB-II for 10 min. While subjects were practicing, the experimenter observed whether they were capable of correctly performing each task.

Three levels of physical activity were considered: no movement, medium (treadmill: 3 km/h, bike: 50 rpm), and high movement (treadmill: 5 km/h, bike: 70 rpm). Since in the case of the stationary bike it was not possible to set the physical activity level for a fixed value during the experiment, we leveraged the training phase prior to each experimental section to let each participant get used to the speeds required during the data collection. Moreover, during each trial, the experimenter monitored whether the participant was deviating more than 5 rpm from the required speed and alerted the participant in case it did.

With respect to the MW levels elicited by MATB-II, two levels were considered, namely, low and high MW according to the task difficulty. In total, six possible combinations of joint MW and physical activity levels were tested. The experiment was then split into six sessions, each one corresponding to one of the six combinations previously described. The order in which each session was executed was counterbalanced among all the participants to avoid any ordering biases.

**Figure 2:**
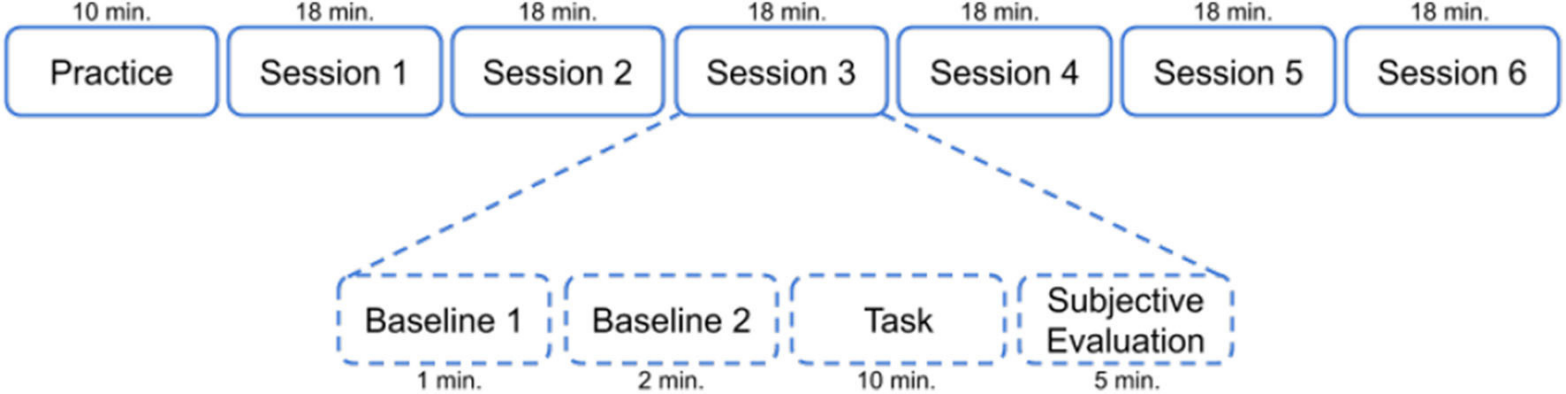
Schematic of the steps executed by a participant during the experiment.

Before each session, data corresponding to two baseline periods were collected. During the first baseline, there were neither physical nor mental activity. Participants were asked to stand still and relax during 60 s. Following this relaxation period, the second baseline was recorded where the subject was asked to start moving according to the corresponding physical activity level assigned to the current session, but without at MW manipulation. Recordings of the second baseline period only began once the activity level reached a stable period and the recording then lasted for 2 min. Lastly, the experimenter gave the joystick to the participant and the 10-min session of combined mental physical effort started. After each task, a 5-min break was given. During this resting period, participants were asked to perform a subjective evaluation corresponding to the past task by filling the NASA-TLX questionnaire. They also reported their perceived fatigue level based on the Borg scale. Overall, the duration of each experimental session comprising the baselines, task, and subjective evaluation was 18 min, and the complete experimental protocol lasted roughly 2 h. Figure 2 summarizes the entire experiment and shows the duration in minutes corresponding to each part of a complete session.

### 3.3. Stimuli

**Figure 3:**
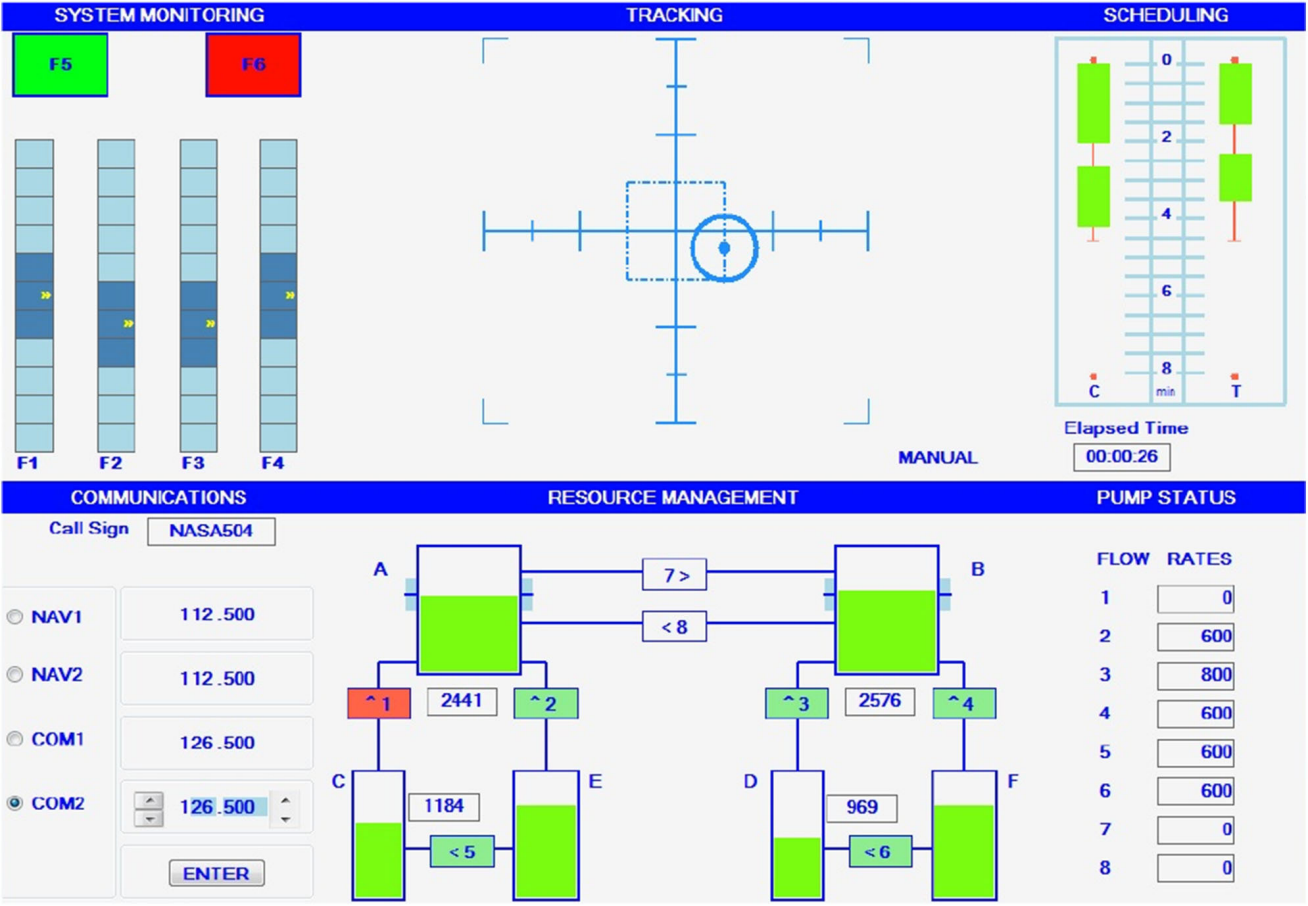
Illustration of the Multi-Task Attribute Battery II (MATB-II) interface (figure obtained from NASA's website https://matb.larc.nasa.gov/).

The MATB-II (Santiago-Espada et al., 2011) was employed for modulating the MW level on the participants. This set of tasks was originally devised to simulate different activities that need to be performed by an aircraft pilot. In our experiment, different mental strain levels are elicited by requiring the subjects to simultaneously perform three of the (four available) tasks involved in MATB-II, namely, system monitoring, tracking, and resource management. Figure 3 shows a screenshot of the MATB-II interface, as seen by the participant. Note the top-right part of the screen was not used for the purposes of this study. An Xbox 360 controller was used to perform the three concurrent activities.

The system monitoring task (see top-left part of Figure 3) requires the participant to monitor four sliders and report deviations from their normal state. The two warning lights (seen as F5 and F6 in the figure) were not used in this study. In their normal states, sliders oscillate around the center position. In their deviation state, sliders start oscillating around the top or the bottom of the panel. Participants had to use the directional pad of the controller to report deviations (one direction was assigned to each slider). When reported, the concerned slider reverted to its normal state. In case the deviated sliders were not reported within 10 s, they were reverted to their normal state and a false alarm was recorded.

The tracking task (top-middle part of Figure 3), in turn, requires the participant to keep a target (a circular aim) within a square bounding box. As the trials progressed, the target started to move randomly. Participants had to use the joystick part of their controller to bring the target back near the center of the square. Lastly, the resource management task (bottom-center part of Figure 3) simulates the control of fuel reservoirs. Participants are asked to control pumps (which are subject to failure during the task) to transfer fuel across 6 reservoirs in order to keep the content levels of two main tanks (A and B) below a certain threshold. In particular, they were instructed to keep the level of the main tanks as close as possible to 2,500 units (this level is indicated by ticks on the sides of tanks A and B). However, fuel gradually depleted from tanks A and B. To keep the tanks at the aimed level, participants could use 8 pumps (labeled 1–8) to transfer fuel between the reservoirs. To activate pumps, participants had to use the second joystick of the controller to move the cursor and “click” on the pumps. When turned on, the pump would turn green. Pumps were configured to fail from time to time. When a pump failed, it turned red and was disabled. Pumps were automatically enabled for use after a while and the participant could resume using it if needed.

Modulation of the MW level relied on changing parameters in MATB-II. For example, for low MW cases, sliding bars speed, aim speed, volume of fuel in the reservoirs, and failure rate of the pumps were set to lower values. In the case of high MW, on the other hand, those parameters were set to larger values.

### 3.4. Subjective Evaluation

As mentioned previously, each experimental session within our protocol included a subjective evaluation. During this step, the NASA Task Load Index (NASA-TLX) questionnaire (Hart and Staveland, 1988) was employed. This set of questions was devised with the main purpose of providing a subjective metric for MW, which is less participant-specific and takes into account different factors resulting in mental strain.

The NASA-TLX questionnaire consists of the evaluation of six factors considered to impact MW, namely, mental demand, physical demand, temporal demand, performance, effort, and frustration. Subjects were asked to perform a self-evaluation of their mental/physical state with respect to each of these six dimensions using a 21-point scale.

In addition to the NASA-TLX questionnaire, we also employed the Borg fatigue scale (Borg, 1982) to assess the participant's fatigue level. They were asked to answer the following question using a scale from 6 to 20: “What physical effort and level of physical fatigue did the last segment impose on you?” We collected fatigue ratings before and after the 5-min break taken at the end of each experimental session.

### 3.5. Devices

In this study, three wireless wearable devices were employed to acquire data from 7 different modalities, as summarized in Table 1. The table also shows the sampling rate used during the data collection for each modality. The open-source software MuLES (Cassani et al., 2015) was utilized in order to allow simultaneous and synchronized acquisition of data streams from all devices. MuLES was also used to generate the synchronized markers indicating the beginning and the end of each phase of the experimental protocol. More details about each device is given below.

**Table 1:** Devices used in the data collection along with the respective acquired modalities and sampling rate.

	**Modality**	**Sampling rate (Hz)**
Enobio	EEG	500
Empatica E4	Skin temperature	4
	Galvanic skin response	4
	Blood volume pulse	64
	Acceleration	32
Bioharness3	ECG	250
	Breathing rate	25
	3-axis acceleration	18

#### 3.5.1. Enobio Headset

EEG data were collected using the 8-channel Neurolelectrics Enobio portable headset (Ruffini et al., 2007). The acquisition sampling rate was set to 500 Hz. Electrode positions according to the 10–20 system were P3, T9, AF7, FP1, FP2, AF8, T10, and P4. References were placed at Fpz and Nz. Since our study involved physical activity, we decided to use wet electrodes on the regions that would be likely affected by sweat during the experiments to avoid signal quality issues (Shu et al., 2019). Therefore, frontal and temporal regions were monitored using wet electrodes, while dry electrodes were used in the parietal region. Figures 1A,B illustrate Enobio's placement on the participant's head during the experiment.

#### 3.5.2. E4 Wristband

The E4 wristband from Empatica was used to sample skin temperature, GSR, BVP, and acceleration at 4, 4, 64, and 32 Hz, respectively. The E4 was placed either on the left or right wrist, according to the participant's preference. In Figure 1A, it is possible to see the E4 positioned on the subject's right wrist. In the case of participants assigned to the treadmill, the E4 position was monitored during the experiment breaks in order to assure skin contact was not lost due to arm movements while running.

#### 3.5.3. BioHarness3

The Bioharness3 acquired ECG, breathing rate, and acceleration at 250, 25, and 18 Hz, respectively. The device was supported by a chest belt containing two wet electrodes, one approximately placed at the tip of the sternum and another on the left side of the chest, both in direct contact with the skin. The length of the belt was carefully adjusted to avoid it from moving during the experiment. In Figure 1B, it is possible to observe the position of BioHarness3 belt across the subject's chest area. Note that this is for visual purposes only and in the actual experimental sessions, the belt was placed in direct contact with the participant's skin.

## 4. Validation of Collected Data

### 4.1. Validation Steps

In this section, we provide an overview of the analysis performed to validate the collected data, both in terms of subjective ratings and psychophysiological recordings. To validate the data obtained from the subjective evaluations, a mixed model analysis of variance (ANOVA) was used for each NASA-TLX dimension and the Borg scale values. As this experiment aimed to test the effect of different experimental conditions on the collected subjective ratings, a repeated measures design was used in order to take into account the within-subject variability on the data. For each aspect considered in the subjective evaluation, MW (with low or high levels) and physical workload (with no, medium, high intensity) were considered as within-subject independent variables, whereas equipment (bike or treadmill) was considered as the between-subject independent variable.

In addition to the ANOVA, we empirically analyzed the changes on the distribution of NASA-TLX dimensions ratings for each different physical strain level. With this analysis, a visual depiction of how different physical activity levels impact the subjective perception of different NASA-TLX factors can be seen. To this end, each rating was first mapped to a binary value (low or high), considering as threshold the respective average rating calculated per subject taking into account all experimental sessions. We then presented for each physical workload level the total number of sessions rated as high for low/high MW sessions. Moreover, we performed the same analysis considering the subjects grouped according to the equipment to manipulate physical strain.

Validation then proceeded by attempting to perform binary classification of MW levels using features commonly reported in the literature and exploring the changes in performance resultant from varying physical workload conditions. It is important to emphasize that as the goal of this paper is to describe the new dataset and validate its use for the purpose intended, achieving state-of-the-art MW level prediction performance is not a priority and exploring the use of new features and/or classifiers is left for future work.

In the following subsection, the features used for benchmark MW classification are described.

### 4.2. Features

For EEG data, signals were downsampled to 250 Hz and bandpass filtered with a bandwidth 1–45 Hz. Wavelet-enhanced Independent Component Analysis (wICA) (Castellanos and Makarov, 2006) was used to reduce the impact ocular and muscular artifacts as it has shown reliable performance on MW assessment across different groups of features (Albuquerque et al., 2019). As the multi-task nature of MATB-II requires frequent changes in gaze position during the experiment, using an enhancement method capable of removing eye-related artifacts is of great importance. Features were then computed from the wICA-enhanced signal over 4-s long epochs with no overlap between consecutive windows. For classifying mental and physical workload levels, classical spectral features were considered, namely power spectral density (PSD) at delta (1–4 Hz), theta (4–8 Hz), alpha (8–12 Hz), beta (12–30 Hz), and gamma (30–45 Hz) frequency sub-bands.

In the case of the physiological modalities collected using the Empatica E4, features were computed over 30-s windows with no overlap between consecutive windows. Mean, median, standard deviation, maximum, and minimum values over the 30-s window were considered as features for classification. In the case of skin temperature, acquired signals were pre-processed to remove high-amplitude peaks artifacts.

For the ECG signal, in turn, a bandpass filter was performed between 5 and 25 Hz to enhance the QRS complex peaks. Visual analysis was then used to remove segments with no clear RR intervals. This was followed by an energy-based QRS detection algorithm (Behar et al., 2014), which is an adaption of the popular Pan & Tompkins algorithm (Pan and Tompkins, 1985). The RR series obtained was further filtered to remove outliers using range-based detection (≥280 and ≤ 1, 500 ms), moving average outlier detection, and a filter based on percent change in consecutive RR values (≤20%), as implemented in Behar et al. (2018). Finally, benchmark time- and frequency-domain heart rate variability (HRV) features were extracted from each session using 5-min windows with a 4-min overlap. The HRV feature set and the window size selection was done based on recommendations made in Camm et al. (1996). The time domain features included mean, standard deviation, and coefficient of variation, while the frequency domain features were high frequency power (HF), normalized HF, low frequency power (LF), normalized LF, very low frequency power, and the ratio between HF/LF.

For the breathing signal, downsampling was first performed from 18 to 6 Hz. A low-pass filter was then applied to remove noise (Chebychev, 2 Hz, 8th order). Following this, descriptive statistical features that include, average, median, standard deviation, minimum, maximum, delta, range, coefficient of kurtosis, and skewness of the signal were calculated. Further, breathing spectrum is sometimes divided into four equally spaced bands between 0 and 0.4 Hz. To explore influence of higher frequency, the spectrum was divided into 5 equally spaced bands between 0 and 1 Hz and power in each of the bands was used as a spectral breathing feature.

## 5. Validation Results and Discussion

### 5.1. Subjective Ratings Analysis

#### 5.1.1. Repeated Measures ANOVA

Table 2 reports the results for multiple mixed model ANOVA^1^ performed on the subjective ratings in terms of partial effect size ($\eta_{p}^{2}$) and *p*-value. We observe that all evaluated subjective ratings were significantly (*p* < 0.001) affected by changes in the type of equipment used to modulate physical strain levels. Similarly, a significant effect (with *p* < 0.05) of MW (represented as MW in the table) manipulation was found for all subjective ratings. Physical workload (PW), in turn, was found to significantly affect (with *p* < 0.05) all subjective ratings except *Performance*. By observing the descriptive statistics of this factor in Table A1 in the Appendix, it can be seen that for all physical activity levels, the average of this NASA-TLX dimension was approximately equal to 15 and 12 for low and high MW sessions, respectively. Interestingly, mental demand ratings are significantly changed by manipulation on physical strain, which indicates that there might be an interplay between physical activity and perceived MW, further confirming the importance of collecting the proposed dataset. No significant interactions were found between MW and equipment, as well as between physical activity and equipment. Finally, no interactions between MW and physical activity were found for all subjective measurements except *Effort*.

**Table 2:** Partial effect size ($\eta_{p}^{2}$) obtained from repeated measures analysis of variance (ANOVA) for subjective ratings (MW, mental workload; PW, physical workload).

		**Equipment**	**MW**	**MW × Equipment**	**PW**	**PW × Equipment**	**MW × PW**
NASA-TLX	Mental demand	0.897^*^	0.555^*^	0.002	0.219^*^	0.006	0.026
	Physical demand	0.857^*^	0.231^*^	0.003	0.723^*^	0.055	0.014
	Temporal demand	0.866^*^	0.602^*^	<0.001	0.350^*^	0.022	0.002
	Performance	0.952^*^	0.679^*^	0.015	0.062	0.005	0.042
	Effort	0.909^*^	0.593^*^	0.013	0.376^*^	0.031	0.066^†^
	Frustration	0.739^*^	0.445^*^	0.022	0.097^†^	0.008	0.041
Borg scale	Before break	0.967^*^	0.437^*^	0.006	0.719^*^	0.056	0.006
	After break	0.961^*^	0.174^†^	0.059	0.619^*^	0.062	0.038

* *p-value ≤ 0.001*,

† *0.001 < p-value ≤ 0.05, NO SYMBOL: p-value > 0.05*.

#### 5.1.2. Distribution of Binary TLX Dimensions

**Figure 4:**
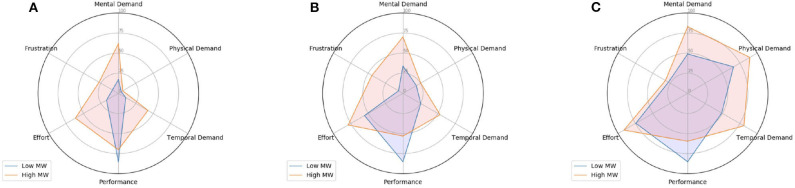
Percentage of high-rated TLX dimensions (using the average value as threshold) per physical activity level. In this case, subjects that performed physical activity using both bike and treadmill are considered. **(A)** No physical activity. **(B)** Medium physical activity. **(C)** High physical activity.

Figure 4 shows the percentage of “high” ratings for each NASA-TLX dimension, considering low and high MW sessions separately (represented in blue and orange, respectively). In order to inspect the effect of changes in physical activity, each radar chart accounts only for data collected under a single physical activity level. Intuitively, we expect high MW sessions to present a higher number of “high” ratings for some of the TLX dimensions such as mental demand. On the other hand, in the case of performance, we suppose a lower number of “high” ratings will be obtained for high MW sessions.

Overall, when comparing the radar charts for data obtained under different physical activity conditions, we notice that the number of high-rated sessions for mental demand increased. Thus, participants found high physical workload sessions more demanding than sessions where there was no physical activity to be performed. We believe this aspect further indicates that mental and physical workload are confounded and this particular relationship should be closely investigated by future research using the described dataset. Moreover, it is possible to observe that the number of “high” ratings for factor *Performance* has not drastically changed when physical workload increased. As we previously highlighted, this was similarly observed in the results obtained by the ANOVA study presented in Table 2. We believe this indicates that, as described by the OFS framework, participants need to increase their physical and mental demand in order to maintain a certain overall performance level.

**Figure 5:**
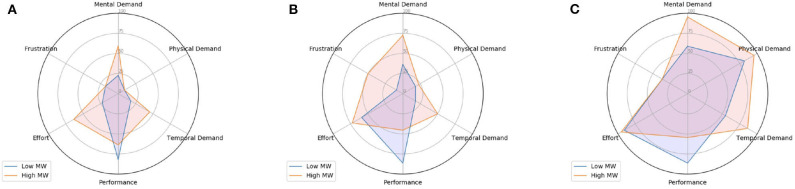
Percentage of high-rated TLX dimensions (using the average value as threshold) per physical activity level. In this case, subjects that performed physical activity using **only the treadmill** are considered. **(A)** No physical activity. **(B)** Medium physical activity. **(C)** High physical activity.

**Figure 6:**
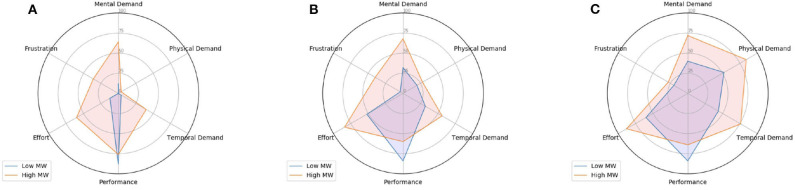
Percentage of high-rated TLX dimensions (using the average value as threshold) per physical activity level. In this case, subjects that performed physical activity using **only the bike** are considered. **(A)** No physical activity. **(B)** Medium physical activity. **(C)** High physical activity.

Moreover, as subjects performed physical activity using either a treadmill (*n* = 22) or a stationary bike (*n* = 26), radar charts for binary TLX ratings are also computed based on the equipment. Different patterns are expected based on equipment used, as for example, participants on the treadmill were holding the controller, thus could not use their arms to help with balance, which could induce changes in cognitive load. Radar charts obtained with treadmill and bike data are shown in Figures 5, 6, respectively. Overall, distributions are found to be indeed different for most of the dimensions/experimental conditions for both equipment. More specifically, by comparing Figures 5A, 6A, it can be seen that for sessions where no physical activity was required and a low MW task was performed, a higher percentage of subjects rated the *Mental Demand* dimension as high for the treadmill case. Despite the fact most of subjects rated this sessions as low physical demand, it is believed that this indicates that as subjects were standing during these sessions, this “extra” physical strain (in comparison to the bike) might be the responsible for increasing the perceived mental demand. Interestingly, in the case high MW sessions performed using a stationary bike, a higher percentage of subjects rated the *Effort* dimension as high.

### 5.2. Classification of MATB-II Mental Workload Levels

Binary MW classification (low vs. high values) was explored using the MATB-II difficulty level as the ground truth. We consider three different cross-validation strategies to train and evaluate classifiers: **(i) Mixed-subjects:** we pool the data from all subjects and use a five-fold cross-validation scheme to split it. This process is repeated 50 times after shuffling the dataset to obtain different cross-validation folds. We report the average performance across the 50 repetitions. **(ii) Intra-subject:** We train one classifier per subject using five-fold cross-validation and report the average performance across all subjects. **(iii) Leave-one-subject-out:** Classifiers are trained with data from all but one subject and then evaluated on data from the subject left out. In this case, we report the average performance across the models obtained when each subject was left out of training. In all cases, Random Forest classifiers with 15 estimators were employed and the performance achieved in terms of the area under the receiving operator curve (AUC) is reported in terms of the average and standard deviation. Notice that it was not possible to apply intra-subject and leave-one-subject-out cross-validation schemes on models trained with ECG and breathing features because the number of data points per subject after feature extraction was considerably low (only two examples per experimental session).

Table 3 presents the classification results obtained using features computed from each modality individually for the no, medium, and high physical workload conditions, as well as for all conditions combined. Overall, we observe that EEG and breathing features presented the best average performance and lowest standard deviation while BVP presented the lowest average performance. Interestingly, classifiers trained on all the conditions combined resulted in the lowest performance, suggesting that a hierarchical classification scheme may be needed where physical workload is first estimated and a PW-specific MW classifier is used. These findings corroborate the hypothesis of an interplay between mental and physical workload.

**Table 3:** Mean and standard deviation of area under the receiving operator curve (AUC) values obtained for binary mental workload classification when considering a model trained with data from all subjects, one model per subject and leave-one-subject-out validation.

**Modality**	**Condition**	**AUC—Mixed subjects**	**AUC—Intra-subject**	**AUC—Leave-one-subject-out**
EEG	No	0.774 ± 0.008	0.823 ± 0.139	0.523 ± 0.073
	Med	0.936 ± 0.004	0.927 ± 0.110	0.511 ± 0.093
	High	0.945 ± 0.004	0.929 ± 0.099	0.518 ± 0.112
	All	0.868 ± 0.004	0.805 ± 0.147	0.500 ± 0.049
Temperature	No	0.679 ± 0.026	0.846 ± 0.258	0.514 ± 0.142
	Med	0.641 ± 0.028	0.830 ± 0.279	0.509 ± 0.125
	High	0.656 ± 0.026	0.787 ± 0.303	0.506 ± 0.122
	All	0.594 ± 0.016	0.632 ± 0.282	0.514 ± 0.069
GSR	No	0.712 ± 0.025	0.882 ± 0.233	0.498 ± 0.144
	Med	0.761 ± 0.027	0.923 ± 0.169	0.522 ± 0.159
	High	0.692 ± 0.026	0.827 ± 0.256	0.557 ± 0.135
	All	0.661 ± 0.015	0.711 ± 0.264	0.519 ± 0.068
BVP	No	0.580 ± 0.029	0.720 ± 0.255	0.512 ± 0.109
	Med	0.624 ± 0.029	0.751 ± 0.258	0.539 ± 0.078
	High	0.584 ± 0.028	0.744 ± 0.249	0.494 ± 0.098
	All	0.562 ± 0.016	0.644 ± 0.183	0.481 ± 0.065
ECG	No	0.778 ± 0.016	-	-
	Med	0.780 ± 0.018		
	High	0.753 ± 0.026		
	All	0.748 ± 0.011		
Breathing	No	0.913 ± 0.011	-	-
	Med	0.892 ± 0.013		
	High	0.903 ± 0.012		
	All	0.865 ± 0.011		

As expected, we observe that individualized models (intra-subject cross-validation) yielded the best performance across all considered modalities. However, this approach requires collecting data and training an MW classifier for each new subject to be monitored, which makes it less practical for real-world applications scenarios. From this perspective, leave-one-subject-out cross-validation appears to be the best strategy to be adopted, since no calibration is required prior to using the obtained MW classifier on new subjects. On the other hand, the overall poor performance of the obtained classifiers under this cross-validation scheme as presented in Table 3 indicates that even though the considered features showed predictive power for MW for individual subjects, they are not robust to cross-subject variability.

In comparison to previous work that also considered MATB-II to modulate mental strain, we observe that the results presented in Table 3 are in-line with the performances previously reported in the literature for experimental setting that considered static subjects. Specifically, Wilson and Russell (2003b) obtained 87.2% using only EEG spectral features, while we were able to achieve an average accuracy of 86.8% when taking into account all the physical workload levels altogether and a model trained using mixed subjects cross-validation (as in Wilson and Russell, 2003b). Similarly to our results, Wilson and Russell (2003b) also observed a decrease in the classification performance when only features computed from physiological modalities were considered. Note that although we obtained similar findings, the study in Wilson and Russell (2003b) only involved seven participants, as opposed to 48 in our case, and different approaches were considered to extract features and design classifiers, rendering the reported performance not directly comparable with results presented herein.

When observing the effect of increasing physical workload on the classification results, it can be seen that, in the case of mixed-subjects and intra-subject cross-validation schemes, EEG-based models obtained better performance when physical strain increased. This might be caused by an increase in the actual perceived MW during the task due to the extra effort not only in performing the physical activity, but also, for example, the increased mental resources used to avoid falling from the treadmill. This, added to the findings presented by Zink et al. (2016), which observed a decrease in the P300 component of EEG data in case subjects were biking in an outdoor environment, provides further evidence of the existence of an interplay between physical activity and perceived MW. For the other physiological features, in turn, the best classification performance was usually achieved in the no/medium PW condition. As the literature on movement artifact removal is more scarce for physiological signals, the findings in Table 3 suggest that new enhancement algorithms may be needed, particularly for the high PW conditions.

### 5.3. Comparing Classification Performance: Bike vs. Treadmill

Recent research has shown that a human's attention to targets is reduced when walking relative to when standing still, due to processing demands produced by visual and inertial stimulation (Ladouce et al., 2019). As such, varying MW prediction capability is hypothesized based on the physical activity equipment used. Table 4 shows the resulting AUC values for binary MW classification when using the treadmill or the stationary bike, as well as with both conditions combined. As can be seen, for all modalities, except ECG and breathing, average AUC values were higher in the treadmill condition. For EEG, these findings corroborate those of Ladouce et al. (2019).

**Table 4:** Mean and standard deviation of area under the receiving operator curve (AUC) values obtained for binary mental workload classification under different signal modalities and physical activity equipment.

**Modality**	**Equipment**	**AUC**
EEG	Treadmill	0.924 ± 0.005
	Bike	0.801 ± 0.007
	All	0.868 ± 0.004
Temperature	Treadmill	0.629 ± 0.022
	Bike	0.626 ± 0.023
	All	0.594 ± 0.016
GSR	Treadmill	0.735 ± 0.022
	Bike	0.666 ± 0.020
	All	0.661 ± 0.015
	Bike	0.534 ± 0.024
	All	0.562 ± 0.016
ECG	Treadmill	0.762 ± 0.017
	Bike	0.773 ± 0.013
	All	0.748 ± 0.011
Breathing	Treadmill	0.875 ± 0.012
	Bike	0.876 ± 0.013
	All	0.865 ± 0.011

### 5.4. Multi-Modal Mental Workload Classification

Lastly, we investigate whether performing MW classification on features computed from different modalities improves the obtained performance. For that, we consider feature-level fusion of EEG, skin temperature, GSR, and BVP features. To synchronize the features between modalities collected with different sampling rates, we average consecutive data points in order to obtain a single data point for each window of 60 s. This process resulted in a total of 10 examples per experimental session, each containing 47 features (32 EEG + 15 from the peripheral signals). In Table 5, we present the resulting AUC for models trained using mixed subjects and leave-one-subject-out cross-validation strategies. Note that we did not include ECG and breathing rate features as this would result in too few data points per subject. Moreover, we did not consider inter-subject cross-validation in this experiment for similar reasons.

**Table 5:** Mean and standard deviation of area under the receiving operator curve (AUC) values obtained for binary mental workload classification simultaneously considering EEG, skin temperature, GSR, and BVP features.

	**AUC—Cross-subject**	**AUC—Leave-one-subject out**
No	0.993 ± 0.006	0.561 ± 0.159
Med	0.998 ± 0.001	0.540 ± 0.253
High	0.998 ± 0.002	0.542 ± 0.217
All	0.995 ± 0.003	0.463 ± 0.115

When comparing the results presented in Table 3 and Table 5, we observe that considering features from multiple modality provided an improvement in the classification performance in almost all the considered cases. Interestingly, we observe that in the case of mixed subjects cross-validation, the multi-modal approach presented improved robustness to an increase physical workload levels, indicating that the simultaneous use of multiple modalities to perform MW assessment might be key to design reliable systems.

### 5.5. Future Research Directions

We believe the WAUC dataset will enable research on several aspects of mobile brain–machine interfaces for practical everyday settings. The following list summarizes the main topics and problems that can be explored within further in-depth analysis of the WAUC dataset:

- Investigate the interplay between physical activity and MW on neural and physiological responses.
- Study the impact of physical strain on the interplay between increased levels of expertise on performing MATB-II and perceived MW (Borghini et al., 2017).
- Develop EEG artifact removal strategies that specifically address noise generated by physical activity for signals collected with low-density devices.
- Devise methods to detect variations on the intensity of MW instead of classifying a specific level.
- Leverage recent developments of deep neural networks to learn representations, which are invariant to subject-specific information in order to improve the performance under real-world scenarios where data from a new subject are not available at training time.
- Develop features tailored to improve robustness to movement artifacts and cross-subject variability.
- Devise models that are able to adapt to changes in the signal-to-noise ratio, as well as to new subjects.

## 6. Conclusions

Operator function state monitoring is critical for optimizing human resources allocation to improve task performance while preserving well-being and safety. In this paper, we focus on the MW component of OFS and propose WAUC, an open multi-modal dataset for assessing the MW under conditions that more closely resemble real-world scenarios. More specifically, the database provides researchers with data from 48 participants, covering seven different modalities measured using off-the-shelf wearable devices, while participants performed six different MW (high/low) vs. physical workload (no/medium/high) tasks, either on a treadmill or a stationary bike. The modalities include electroencephalogram, ECG, breathing rate, skin temperature, GSR, BVP, and 3-axis accelerometry. The MATB-II assessment was used to modulate MW level. Each participant also provided subjective workload ratings using the NASA-TLX questionnaire, as well as Borg fatigue scale ratings.

Besides describing the experimental procedure, detailed validation analysis of the recorded subjective ratings and neuro-physiological signals is also provided, along with a number of research directions that can be followed from the WAUC dataset. The database is available to the research community at: http://musaelab.ca/resources/.

## Data Availability Statement

The raw data supporting the conclusions of this article is available at http://musaelab.ca/resources/, without undue reservation.

## Ethics Statement

Written informed consent was obtained from the individual for the publication of any potentially identifiable images or data included in this article.

## Author Contributions

All authors: experimental design. IA, AT, MP, J-FG, DL, ST, and TF: writing and reviewing. IA, MP, AT, and RC: statistical analysis and programming. ST and TF: funding and supervision.

## Conflict of Interest

J-FG and DL were employed by the company Thales Research and Technology Canada. The remaining authors declare that the research was conducted in the absence of any commercial or financial relationships that could be construed as a potential conflict of interest.

## Footnotes

^1^Although the residuals from the obtained model are not Gaussian according to the Kolmorov–Smirnov test (with significance level of 95%), we observed that the histograms of residuals present a shape similar to a Gaussian distribution for all the considered subjective measures, except Frustration.

## Appendix

**Table A1:** Subjective ratings descriptive statistics (mean and standard deviation) for subjects that used the treadmill (top rows) and bike (bottom rows) during the experiment.

		**No PW**		**Medium PW**		**High PW**	
		**Low MW**	**High MW**	**Low MW**	**High MW**	**Low MW**	**High MW**
**Treadmill**							
NASA-TLX	Mental demand	8.41 ± 5.66	11.27 ± 6.63	10.36 ± 5.09	13.41 ± 5.92	11.23 ± 5.07	15.50 ± 3.56
	Physical demand	4.32 ± 5.05	5.36 ± 5.66	8.23 ± 4.89	9.00 ± 5.12	14.59 ± 4.75	15.41 ± 4.54
	Temporal demand	7.23 ± 6.05	10.27 ± 6.91	8.41 ± 4.59	11.68 ± 5.91	11.41 ± 5.48	15.05 ± 4.34
	Performance	16.36 ± 4.10	12.27 ± 4.12	14.95 ± 4.13	11.05 ± 3.90	15.14 ± 4.11	12.14 ± 4.28
	Effort	8.86 ± 5.76	11.68 ± 5.56	11.45 ± 4.64	13.64 ± 5.19	13.91 ± 4.51	16.36 ± 3.55
	Frustration	6.64 ± 6.64	8.64 ± 6.77	6.59 ± 5.75	10.32 ± 7.17	7.86 ± 6.56	10.18 ± 6.73
Borg Scale	Before break	8.05 ± 2.77	13.59 ± 2.77	10.09 ± 3.04	11.36 ± 2.98	8.86 ± 3.21	14.77 ± 2.16
	After break	8.95 ± 3.20	12.64 ± 2.85	9.27 ± 2.37	10.00 ± 2.62	8.64 ± 3.33	12.95 ± 3.11
**Bike**							
NASA-TLX	Mental demand	6.40 ± 3.44	10.12 ± 4.00	9.00 ± 4.71	11.36 ± 4.70	9.24 ± 4.99	12.60 ± 4.56
	Physical demand	3.04 ± 2.59	3.44 ± 3.22	7.96 ± 3.79	8.84 ± 4.79	11.00 ± 5.40	12.92 ± 4.81
	Temporal demand	5.44 ± 3.48	9.20 ± 4.02	8.20 ± 4.53	11.20 ± 5.37	9.56 ± 4.84	12.80 ± 4.86
	Performance	17.20 ± 4.01	12.60 ± 4.43	15.20 ± 4.07	12.72 ± 4.43	15.08 ± 4.56	12.88 ± 4.56
	Effort	6.48 ± 4.11	11.20 ± 4.05	10.48 ± 4.48	12.52 ± 5.12	11.12 ± 5.37	13.40 ± 4.44
	Frustration	4.28 ± 3.25	6.88 ± 5.09	6.00 ± 4.12	9.12 ± 6.02	8.60 ± 5.39	8.56 ± 4.71
Borg Scale	Before break	7.20 ± 1.58	12.40 ± 2.69	10.36 ± 2.02	11.24 ± 2.73	8.56 ± 2.53	12.92 ± 2.66
	After break	6.96 ± 1.24	11.16 ± 2.58	8.92 ± 2.10	10.20 ± 2.71	8.00 ± 2.18	11.32 ± 2.58
